# Identification of key biomarkers associated with immune cells infiltration for myocardial injury in dermatomyositis by integrated bioinformatics analysis

**DOI:** 10.1186/s13075-023-03052-4

**Published:** 2023-04-28

**Authors:** Yue Zhang, Linwei Shan, Dongyu Li, Yinghong Tang, Wei Qian, Jiayi Dai, Mengdi Du, Xiaoxuan Sun, Yinsu Zhu, Qiang Wang, Lei Zhou

**Affiliations:** 1 Department of Cardiology, The First Affiliated Hospital of Nanjing Medical University, Nanjing, China; 2 Department of Rheumatology, The First Affiliated Hospital of Nanjing Medical University, Nanjing, China; 3 Department of Radiology, The First Affiliated Hospital of Nanjing Medical University, Nanjing, China

**Keywords:** Dermatomyositis, Myocardial injury, Bioinformatic, Immune cells infiltration, Biomarker

## Abstract

**Background:**

Dermatomyositis (DM) is an acquired autoimmune disease that can cause damage to various organs, including the heart muscle. However, the mechanisms underlying myocardial injury in DM are not yet fully understood.

**Methods:**

In this study, we utilized publicly available datasets from the Gene Expression Omnibus (GEO) database to identify hub-genes that are enriched in the immune system process in DM and myocarditis. Weighted gene co-expression network analysis (WGCNA), differentially expressed genes (DEGs) analysis, protein–protein interaction (PPI), and gene ontology (GO) analysis were employed to identify these hub-genes. We then used the CIBERSORT method to analyze immune cell infiltration in skeletal muscle specimens of DM and myocardium specimens of myocarditis respectively. Correlation analysis was performed to investigate the relationship between key genes and infiltrating immune cells. Finally, we predicted regulatory miRNAs of hub-genes through miRNet and validated their expression in online datasets and clinical samples.

**Results:**

Using integrated bioinformatics analysis, we identified 10 and 5 hub-genes that were enriched in the immune system process in the database of DM and myocarditis respectively. The subsequent intersections between hub-genes were IFIT3, OAS3, ISG15, and RSAD2. We found M2 macrophages increased in DM and myocarditis compared to the healthy control, associating with the expression of IFIT3, OAS3, ISG15, and RSAD2 in DM and myocarditis positively. Gene function enrichment analysis (GSEA) showed that IFIT3, OAS3, ISG15, and RSAD2 were mainly enriched in type I interferon (IFN) signaling pathway, cellular response to type I interferon, and response to type I interferon. Finally, we verified that the expression of miR-146a-5p was significantly higher in the DM with myocardial injury than those without myocardial injury (*p* = 0.0009).

**Conclusion:**

Our findings suggest that IFIT3, OAS3, ISG15, and RSAD2 may play crucial roles in the underlying mechanism of myocardial injury in DM. Serum miR-146a-5p could be a potential biomarker for myocardial injury in DM.

## Background

Dermatomyositis (DM) is an acquired autoimmune disease in which skeletal muscle is targeted by the immune system, which is a subset of idiopathic inflammatory myopathy (IIM) [1]. DM is characterized primarily by muscle inflammation, proximal muscle weakness, and cutaneous involvement. Additionally, the disease may present with extra-muscular symptoms affecting various organs including the heart, joints, lungs, and gastrointestinal tract [2].

Several studies have identified cardiovascular involvement in DM is a feared threat to prognosis and a frequent cause of death in several studies [3–5]. Due to the structural and functional similarities between skeletal and cardiac muscle, it is supposed that what affects skeletal muscle may be related to damage to cardiac muscle. In autopsy studies, myocarditis was the most common pathologic features [6, 7], and previous imaging studies also indicate inflammation as an underlying pathology [8]. In different animal models of IIM, the myocardium shows an inflammation similar to that of skeletal muscle morphologically [9].

Despite the accumulating evidence linking DM and cardiovascular involvement, the extent of this link and the underlying mechanisms are not yet fully understood. Moreover, specific recommendations for the treatment of patients with DM and cardiovascular involvement are lacking. Thus, it is essential to explore the molecular characteristics and mechanisms of myocardial injury in DM before developing screening recommendations and treatment strategies for patients with DM and myocardial injury. Over the past decades, gene microarray technology together with integrated bioinformatic analyses has been performed to provide tremendous assistance in identifying novel key genes related to various diseases. In this study, we identified the co-expression genes related to the immune system process between DM and myocarditis, explored the association between these genes and immune cells infiltrating skeletal muscle or myocardium, and predicted regulatory miRNAs of the hub-genes. Finally, we verified our results through online datasets and clinical samples. With the above approaches, it is hoped that our results may provide a preliminary insight into the mechanism of myocardial injury in DM and a search for possible biomarkers.

## Methods

### Data sources

Series matrix files and platform information of GSE1551, GSE48280, GSE5370, GSE128470, GSE1145, GSE35182, and GSE147517 were obtained from the National Center Biotechnology Information Gene Expression Omnibus (NCBI-GEO) (https://www.ncbi.nlm.nih.gov/geo). We selected 13 DM patients and 10 normal individuals, 5 DM patients, and 5 normal individuals, and 5 DM patients and 4 normal individuals from GSE1551, GSE48280, and GSE5370, respectively, and the samples were all skeletal muscle biopsy specimens. We merged the GSE1551, GSE48280, and GSE5370 and used the SVA software package to correct the bath. Then, we used principal component analysis (PCA) [10] to evaluate the results of the correction. Finally, we obtained a normalized gene expression matrix file containing 42 samples (23 DM patients, and 19 normal individuals). We chose samples of myocardium obtained from 11 normal individuals and 7 inflammatory cardiomyopathy patients due to viral myocarditis in GSE1145. In the section of validation, we chose GSE128470 which includes 12 samples of muscle obtained from normal individuals and 12 samples from DM patients. Furthermore, we used GSE35182 to validate the expression of genes between chronic myocarditis and normal mice; there are 6 mice per group. Finally, we validated the predicted target miRNAs expression in GSE147517 comprising 5 myocarditis and 5 normal individuals serum specimens.

### Construction of weighted gene co-expression network analysis

The WGCNA package in R was utilized to build a co-expression network targeting the top 5000 genes with median absolute deviation [11, 12]. The R function pickSoftThreshold was used to calculate the soft thresholding power *β*, to which co-expression similarity is raised to calculate adjacency. Then, we converted the adjacency into a topological overlap matrix (TOM) to measure the network connectivity of genes. Genes with similar patterns were clustered into the same modules (minimum size = 30) using average linkage hierarchical clustering, which were represented by branches and different colors of the cluster tree, constructed module relationships, calculation of the correlation between gene modules and phenotypes, and the modules related to clinical traits were identified. Gene significance (GS) and module membership (MM) were calculated to relate modules to clinical traits. Finally, the highly correlated module was analyzed to explore its core genes and potential roles.

### Identification of differentially expressed genes

The limma package in R (Version 4.0.3) software was utilized to screen DEGs between inflammatory cardiomyopathy patients and normal controls [13, 14]. The differentially expressed genes were screened under the condition of |log FC|> 1 and adjusted *P* value < 0.05.

### Evaluation of immune cell infiltration

CIBERSORT [15] is a deconvolution algorithm for analyzing gene expression data and uses a gene expression signature for characterizing the proportion of each immune cell type. We performed immune infiltration by using the CIBERSORT.R script downloaded from the CIBERSORT website (https://cibersortx.stanford.edu/). We used the original CIBERSORT gene signature file LM22, which defines 22 immune cell subtypes, to calculate the proportion of 22 immune cells in DM and inflammatory cardiomyopathy patients. Then, we used the “ggplot2” package [16] to draw violin diagrams to visualize the differences compared to normal controls in immune cell infiltration. We also calculated the Spearman correlation coefficient between identified hub-genes with infiltrating macrophages M2.

### Functional annotation

Metascape (http://Metascape.org/gp/index.html) is a free web-based analytics tool for comprehensive gene annotation and analysis resources, combining a GO and KEGG pathway enrichment analysis search to leverage over 40 independent knowledge bases [17–19]. To understand the function of these core genes and DEGs, we used metascape to perform GO and KEGG pathway enrichment analyses.

### Protein–protein interaction network analysis

Search Tool for the Retrieval of Interacting Genes (STRING) (Version 11.3, https://cn.string-db.org/) is a useful online tool dedicated to analyzing the functional protein association networks [20–22]. The core genes and DEGs were mapped to the STRING database, and only the experimentally validated interactions with a combined score > 0.4 were selected as significant. Subsequently, the PPI network was visualized by Cytoscape software (version 3.5.1) (www.cytoscape.org/) [23]. The plug-in cytoHubba in Cytoscape was used to screen the hub-genes from the PPI network, and in our study, the top ten genes were identified as hub-genes [24].

### Prediction of potential hub-gene related target miRNAs

We used miRNet (www.mirnet.ca/) [25], a tool that integrates data from 11 different miRNA databases, to predict regulatory miRNAs of the common hub-genes.

### Subjects

A total of 10 DM patients were enrolled from the First Affiliated Hospital of Nanjing Medical University between January 2020 and January 2021. We divided these patients into two groups: DM with myocardial injury (*n* = 5) and DM without myocardial injury (*n* = 5) based on the results of the cardiac magnetic resonance examination. The inclusion criteria were as follows: (1) age > 18 years old, (2) all the patients fulfilled the 1975 Bohan and Peter criteria for dermatomyositis [26], and (3) patients who underwent cardiac magnetic resonance examination during hospitalization. The exclusion criteria were as follows: (1) interstitial lung disease, (2) previous history of cardiac disease, (3) tumor, (4) renal insufficiency, and (5) surgery within the six previous months. Ethical approval was obtained for our single-center cross-sectional study, and the need to obtain informed consent was waived (2020-SR-228).

### Sample collection and RNA isolation

Venous blood samples were collected into EDTA tubes from DM patients within 24 h before and after the cardiac magnetic resonance examination. Serum was obtained at room temperature of 3000 rpm for 5 min and frozen at – 80 °C for further use. Isolation of total RNA including miRNA was performed from serum using the miRNeasy Serum/Plasma Advanced Kit (Qiagen, Germantown, MD, USA) according to the manufacturer’s protocol. During the extraction, 3.5 μL of miRNeasy Serum/Plasma Spike-In Control (1.6 × 10^8^ copies/μL of the C. elegans miR-39 miRNA mimic) was added to each sample as an internal control. RNA quantity and quality were evaluated using the Nanodrop ND-2000 spectrophotometer (Nanodrop Technologies, Wilmington, DE). Directly after isolation, RNA was subjected to the reverse transcription process.

### Reverse transcription reaction and real-time quantitative PCR (RT-qPCR)

The expression levels of miR-27b-3p, miR-130a-3p, miR-1-3p, miR-133a-3p, miR-16-5p, and miR-146a-5p were measured using the Bulge-Loop™ miRNA qRT-PCR Starter Kit (one RT primer and a pair of qPCR primers for each set) designed specifically by RiboBio (Guangzhou, China) in accordance with the manufacturer’s instructions. The average expression levels of serum miRNAs were normalized against cel-miR-39 (Qiagen, Germantown, MD, USA). The fold-change (FC) for each miRNA relative to the control was calculated using the 2^−ΔΔCT^ method [27]. The efficiency was tested before using the 2^−ΔΔCT^ method, and the results were collected from the same experiment in three replicates.

### CMR

All participants underwent CMR imaging on a 3.0 T whole-body scanner (MAGNETOM Skyra, Siemens Healthcare, Erlangen, Germany) with an 18-channel phase-array coil using ECG gating. Late gadolinium enhancement (LGE) images were acquired 8–15 min after intravenous administration of gadolinium-DTPA (Magnevist, Bayer, Berlin, Germany) at a dose of 0.2 mmol/kg in short-axis stack using a phase-sensitive inversion-recovery (PSIR) gradient echo sequence. Typical parameters of motion-corrected basal, mid, and apical level of LV short-axis Modified Look-Locker inversion-recovery (MOLLI) T1 mapping sequence with a 5(3)3 scheme before and 15–20 min after intravenous contrast agent injection.

The native and post-contrast T1 values of the myocardium were measured on a region of interest at the myocardial septum of mid-ventricular short-axis slice by two experienced blinded investigators in consensus with CMR using commercial software (CVI42, Circle Cardiovascular Imaging, Calgary, Canada). The presence of LGE was defined with a signal intensity level increase of more than five S.D. of remote myocardium on the all short-axis contrast images from base to apex [28]. ECV was calculated by the following equation: ECV = (1—HCT) $\frac{\frac{1}{\text{post contrast T1 myo}} - \frac{1}{\text{native T1 myo}}}{\frac{1}{\text{post contrast T1 blood}} - \frac{1}{\text{blood T1 myo}}}$.

Determination of hematocrit (HCT) and calculation of ECV were completed within 24 h after CMR scanning. ECV is a marker of myocardial tissue remodeling and provides a physiologically intuitive unit of measurement. Normal ECV values of 24% ± 3 (3.0 T) have been reported in healthy individuals [29]. In our study, we divide patients with LGE positive and ECV > 25% into DM with myocardial injury group [30].

### Statistical analysis

All statistical analyses were performed in the R language (Version 4.0.3). All statistical tests were bilateral, and adjusted *P* value < 0.05 was statistically significant.

## Results

### Bioinformatic analysis workflow

**Fig. 1:**
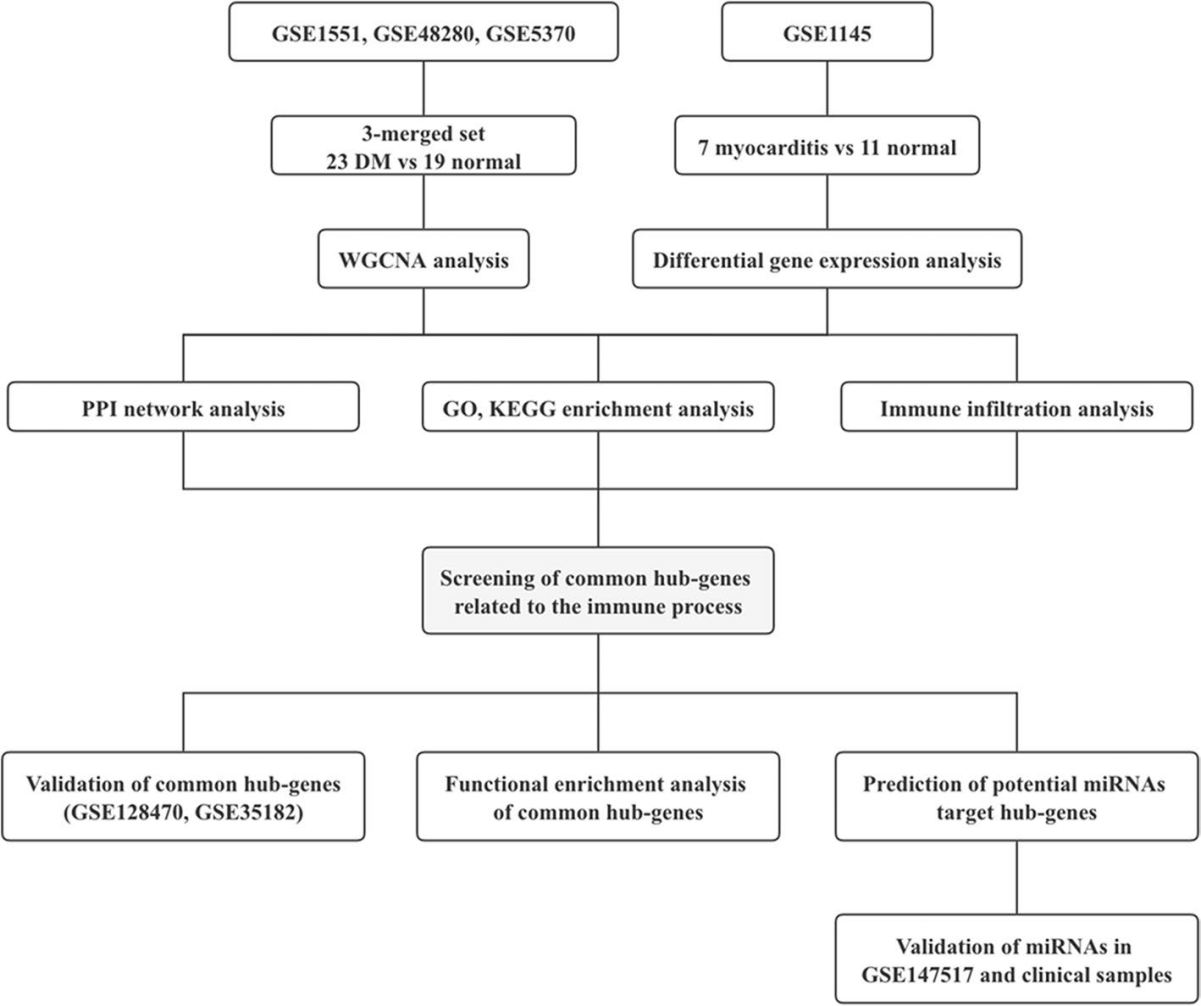
Flowchart of this study

Our workflows are shown in Fig. 1. First, we found key modules related to DM using WGCNA and discovered hub-genes associated with the immune system process through PPI and GO/KEGG enrichment analysis of core genes in key modules. Second, we searched DEGs between myocarditis and normal myocardium, and the same PPI and GO/KEGG enrichment analysis was used to screen for hub-genes associated with the immune system process in myocarditis. The common hub-genes related to immune processes in both DM and myocarditis were obtained by intersecting the above two sets of hub-genes. The function of these common hub-genes and their relationship with immune infiltrating cells were then investigated. Finally, we used miRNet to predict regulatory miRNAs of the common hub-genes and validated their expression in online datasets and clinical samples.

### Identification and analysis of key module of DM by WGCNA

**Fig. 2:**
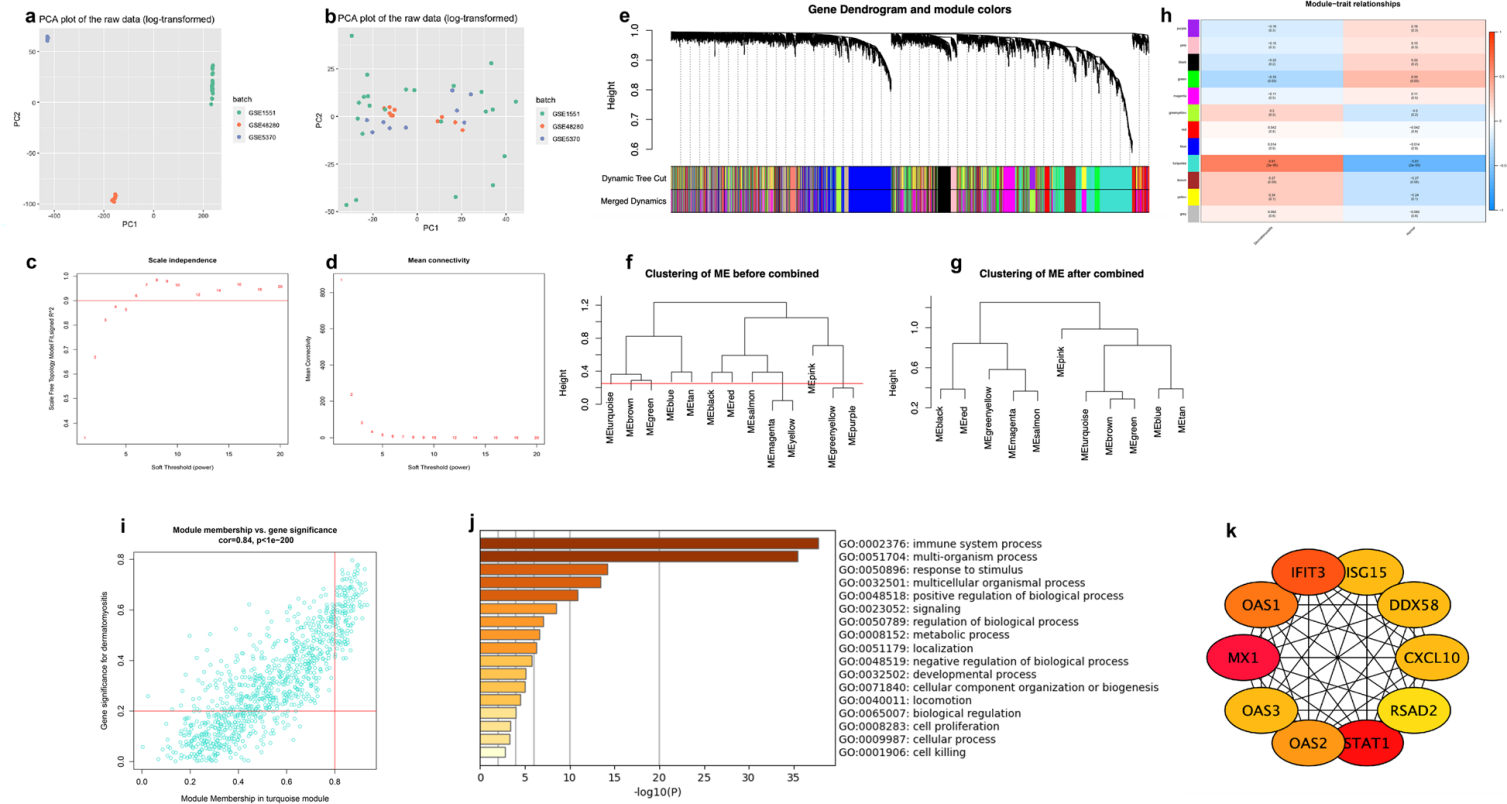
Screening of hub-genes related to the immune system process in DM. **a** PCA before the batch correction of three datasets. **b** PCA after the batch correction of three datasets. **c** Analysis of the scale-free fit index for various soft-threshold powers; the red line was set at 0.90. **d** Analysis of the mean connectivity for various soft-threshold powers. **e** Clustering dendrogram of genes in the co-expression network. **f** Clustering of all modules, the red line indicates the height cutoff (0.25). **g** Cluster of merged modules. **h** Identification of weighted gene co-expression network modules associated with DM. **i** The MM versus GS scatter plot of the turquoise module. **j** Functional annotation of the 120 hub-genes involved in the turquoise module. **k** The major PPI network analysis of the top 10 hub-genes from 120 hub-genes through cytoHubba software

To construct a gene co-expression network, the data of three series matrix files were downloaded from the GEO database. A principal component analysis was performed to visualize the grouping of read counts and identify batch effects. Then, we merged these three datasets into one dataset and corrected the batch using the SVA software package. Figure 2b shows that the inter-batch variations are effectively removed after data normalization. Finally, a normalized gene expression matrix file containing 42 samples (23 DM, 19 healthy people) was obtained. We calculated the median absolute deviation for each gene, sorted the values from large to small, and then selected the top 5000 genes for WGCNA. The expression data map of these 5000 genes was constructed into a gene co-expression network using the WGCNA package in R software. By setting the soft-threshold power as 6 (scale-free R2 = 0.92, slope = -2.15; Fig. 2c, d) and cut height as 0.25, we acquired 12 modules (Fig. 2e–g), genes that cannot be included in any module were added to the grey module and rejected in subsequent analyses.

From the heatmap of module-trait correlations, we found that the turquoise module was the most highly correlated with DM (correlation coefficient = 0.61, *p* = 2e − 05; Fig. 2h). We analyzed the correlation between module membership and gene significance in the turquoise module. The results showed that module membership in the turquoise module (*r* = 0.84, *p* = 1e − 200) was significantly correlated with gene significance for DM. The turquoise module contained 954 genes; 120 genes were identified as core genes with high MM (> 0.8) and GS (> 0.2) values (Fig. 2i). We found that the core genes were the most enriched in the immune system process through GO and KEGG enrichment analysis on the metascape website (Fig. 2j). In the major PPI network analysis of top 10 hub-genes from 120 hub-genes through the cytoHubba software, the shade of the node’s color reflects the degree of connectivity (Fig. 2k); all 10 hub-genes were enriched in the immune system process.

### Identification and analysis of DEGs in myocarditis database

**Fig. 3:**
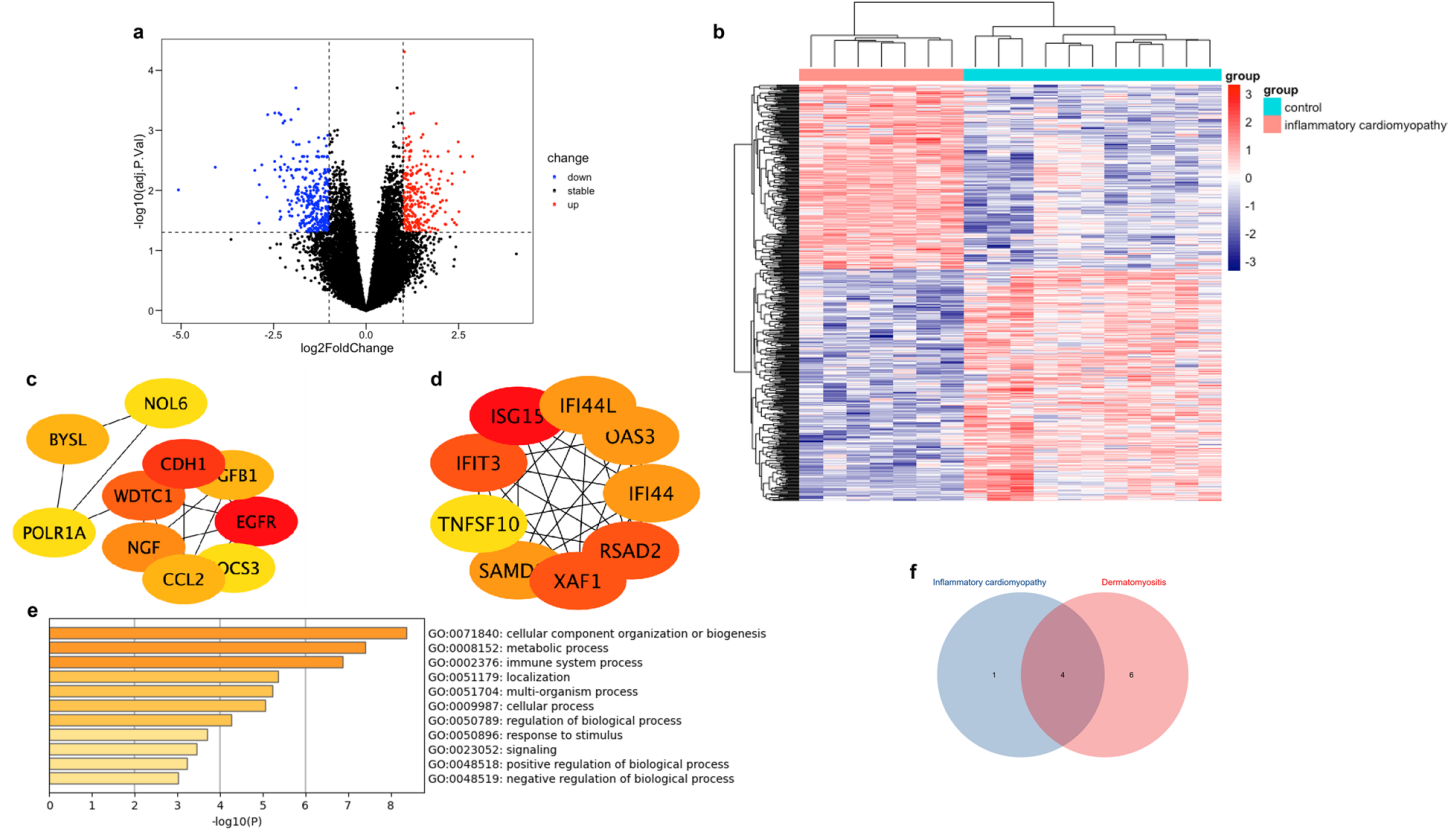
Screening of hub-genes related to the immune system process in inflammatory cardiomyopathy. **a** The volcano plot of DEGs in inflammatory cardiomyopathy. **b** Heat map clustering of the DEGs between normal controls and inflammatory cardiomyopathy patients. **c** The major PPI network analyzing of top 10 hub-genes from downregulated genes. **d** The major PPI network analyzing of top 10 hub-genes from upregulated genes. **e** Functional annotation of the 20 hub-genes contained in **c** and **d**. **f** A Venn diagram showing the number of commonly expressed genes between hub-genes related to the immune system process in inflammatory cardiomyopathy and DM

We identified 570 DEGs composed of 253 upregulated and 317 downregulated genes by using the limma package. The volcano plot (Fig. 3a) showed DEGs between inflammatory cardiomyopathy and normal controls. We imported 253 upregulated and 317 downregulated genes into STRING database to construct the PPI network complex respectively; we then used cytoHubba App in cytoscape to examine hub-genes based on the “degree” algorithm. The genes ISG15, IFIT3, XAF1, RSAD2, IGF1, OAS3, IFI44, SAMD9L, IFI44L, and TLR3 were the top ten upregulated genes (Fig. 3d), and the genes EGFR, CDH1, WDTC1, NGF, BYSL, CCL2, TGFB1, SOCS3, POLR1A, and NOL6 were the top ten downregulated genes (Fig. 3c). GO and KEGG enrichment analyses of the above 20 hub-genes were performed using the metascape (Fig. 3e). Among them, IFIT3, OAS2, ISG15, XAF1, and RSAD2 were enriched in the immune system process. The subsequent intersection between hub-genes related to the immune system process in inflammatory cardiomyopathy and hub-genes in DM using the VennDiagram package yielded 4 common DEGs: IFIT3, OAS3, ISG15, and RSAD2 (Fig. 3f).

### Evaluation of immune cell infiltration and immune cell correlation analysis

**Fig. 4:**
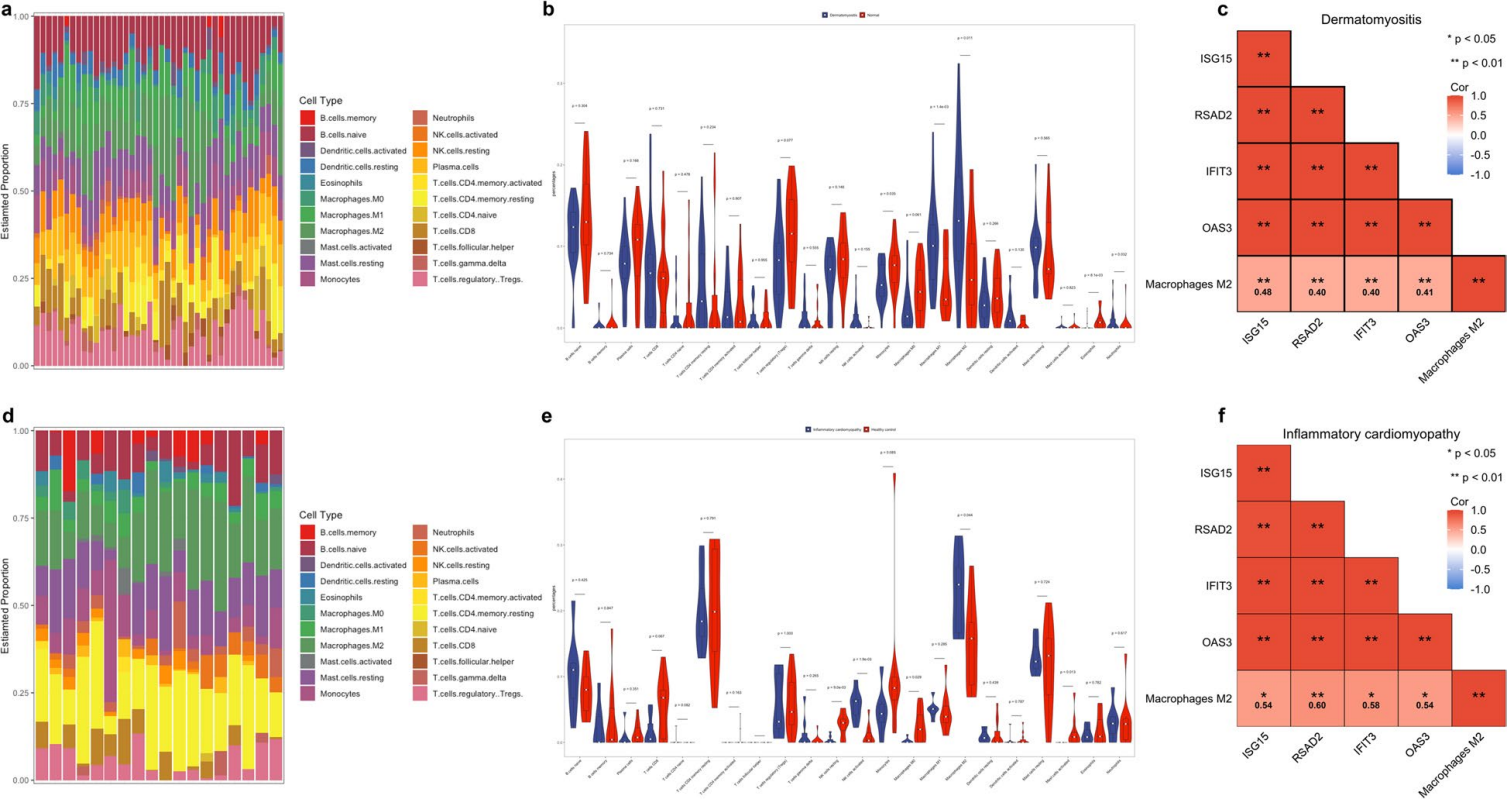
Immune cell infiltration analysis. **a** Heat map of relative proportions of 22 infiltrated immune cells in patients with DM. **b** Violin chart of the abundance of each type of immune cell infiltration in DM and control groups. **c** The correlation analysis of hub-genes and M2 macrophages in DM. **d** Heat map of relative proportions of 22 infiltrated immune cells in patients with inflammatory cardiomyopathy. **e** Violin chart of the abundance of each type of immune cell infiltration in inflammatory cardiomyopathy and control groups. **f** The correlation analysis of hub-genes and M2 macrophages in inflammatory cardiomyopathy

CIBERSORT analytical tool calculated the fractions of 22 types of leukocyte subpopulations in myocardial tissue and skeletal muscle samples respectively, including naïve B cells, memory B cells, plasma B cells, CD8 + T cells, CD4 + naïve T cells, CD4 + memory resting T cells, CD4 + memory activated T cells, follicular helper T cells, regulatory T cells (Tregs), gamma delta T cells, resting natural killer (NK) cells, activated NK cells, monocytes, M0, M1, and M2 macrophages, resting and activated myeloid dendritic cells, resting and activated mast cells, eosinophils, and neutrophils. The violin plot of the immune cell infiltration difference showed that M1 macrophages in the DM were higher than that in the control group, while M2 macrophages in both the DM and the inflammatory cardiomyopathy group were higher than that in the control group (Fig. 4). Then, the spearman correlation coefficient between hub-genes and the infiltration level of the immune cell was calculated. As a result, M2 macrophages were positively associated with the expression of IFIT3, OAS3, ISG15, and RSAD2 in patients with inflammatory cardiomyopathy and dermatomyositis, respectively (Fig. 4c, f).

### Validation hub-genes with GEO databases

**Fig. 5:**
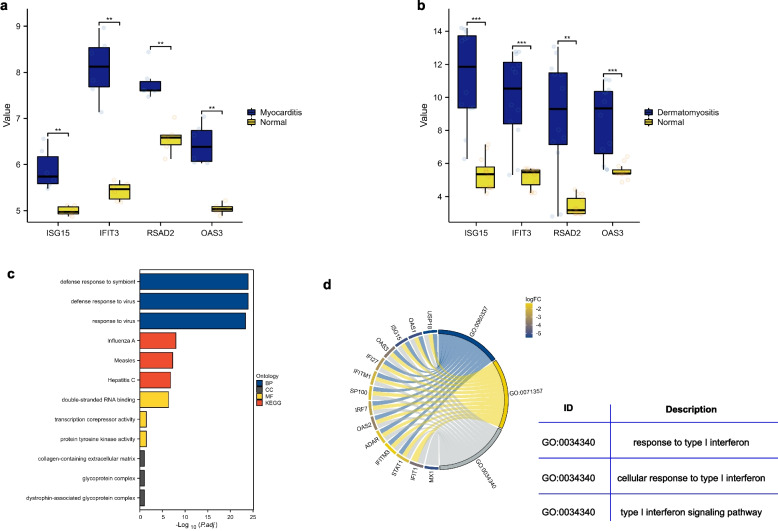
Validation and functional enrichment analysis of hub-genes in GEO databases. **a** Validation of hub-genes in GSE35182. **b** Validation of hub-genes in GSE128470. **c** The enriched biological process, cell component, molecular function, and KEGG pathways of DEGs (DEGs between two clusters divided by the expression of common hub-genes). **d** Circos plot to indicate the relationship between genes and biological process terms

To further validate the expression of IFIT3, OAS3, ISG15, and RSAD2 in myocardial tissue and skeletal muscle tissue, we selected GSE128470 and GSE35182 as testing datasets. As shown in Fig. 5a, the expression levels of IFIT3, OAS3, ISG15, and RSAD2 were verified in myocardial tissue between myocarditis mice and normal control. Then, the expression levels of IFIT3, OAS3, ISG15, and RSAD2 were also significantly higher in DM skeletal muscle tissues than in the normal controls (Fig. 5b).

### Functional enrichment analysis of IFIT3, OAS3, ISG15, and RSAD2

To further explore the potential function of the common hub-genes, GO/KEGG enrichment analysis was performed. We divided the samples from the GSE128470 dataset into a high-expression group and a low-expression group according to the median expression level of the common hub-genes and identified DEGs between the two clusters. As shown in Fig. 5c, d, DEGs were mainly enriched in type I interferon signaling pathway, cellular response to type I interferon, and response to type I interferon.

### Prediction and validation of potential miRNAs targeting hub-genes

**Fig. 6:**
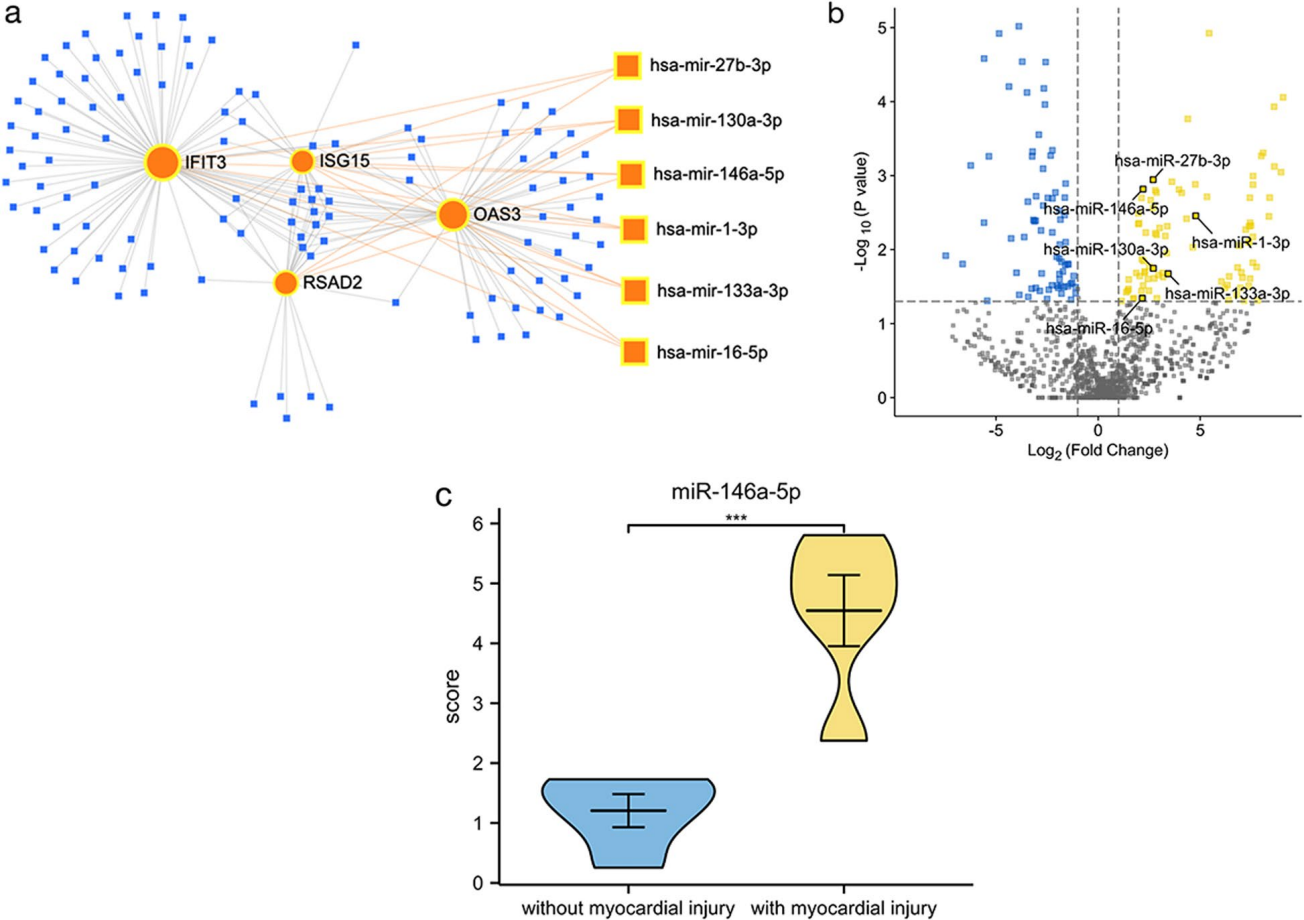
Screening and validation of potential miRNAs targeting hub-genes. **a** An Interaction network of four hub-genes and potential miRNAs-targeted. **b** The volcano plot of DE-miRNAs between myocarditis and normal control in GSE147517. **c** Validation of miR-146a-5p expression in the serum of patients with DM

We applied the miRNet database to screen the targeted miRNAs of ISG15, IFIT3, RSAD2, and OAS3. As illustrated in Fig. 6a, a total of 122 miRNAs were predicted, 23 with association with three or more genes, six of which were confirmed to be upregulated in serum exosomes of patients with myocarditis than normal control (Fig. 6b).

### Validation of miRNAs in serum samples

To verify the clinical application potential of miR-27b-3p, miR-130a-3p, miR-1-3p, miR-133a-3p, miR-16-5p, and miR-146a-5p, comparative analysis of these 6 microRNAs between DM with myocardial injury and DM without myocardial injury was performed. The baseline demographic and clinical characteristics of 10 DM patients were shown in Table 1. The level of miR-146a-5p expression was statistically significantly higher in the DM with myocardial injury group than the other group (*p* = 0.0009) (Fig. 6c).

**Table 1:** The characteristics of DM patients between two groups with and without myocardial injury

	DM with myocardial injury (*n* = 5)	DM without myocardial injury (*n* = 5)	*p* value
Age, year	47.80 ± 6.61	52.00 ± 9.43	0.441
Female (*n*, %)	5 (100%)	5 (100%)	1
Disease duration, month	6.20 ± 4.66	3.20 ± 2.77	0.251
cTNT, ng/L	80.85 ± 14.95	168.21 ± 125.36	0.347
CKMB, U/L	70.94 ± 128.15	218.36 ± 190.35	0.189
Pro-BNP, pg/ml	83.98 ± 70.58	198.93 ± 164.78	0.246

## Discussion

Although the cause of DM pathogenesis remains unclear, previous studies have established the existence of myocardial injury in DM. Autopsy studies indicate that myocarditis is the most frequent pathological manifestation [3, 31]. Some serological biomarkers may serve as a screening tool for myocardial injury in DM such as cTNI and GDF-15 [32, 33]. Despite this, little research has explored the genetic underpinnings of myocardial injury in DM. Our study has identified several common hub-genes that are associated with both the immune process of myocarditis and DM. These genes may play a role in the onset of myocardial injury in DM. By identifying miRNAs that target these genes and validating their potential, we have found miR-146a-5p to be a promising biomarker for detecting myocardial injury in DM.

Autopsy studies have revealed that myocarditis is the predominant pathological feature of myocardial injury in DM, with the immune system thought to play a pivotal role in the development of both conditions. Based on the above two reasons, we used an integrated bioinformatics analysis to screen hub-genes related to the immune system process in DM and myocarditis respectively. The intersection of these two sets of genes represents a group of common hub-genes that are believed to be linked to myocardial injury in DM. This approach has been successfully applied in a variety of biological contexts to identify common risk genes and mechanisms associated with multiple disease phenotypes [34–36]. In our study, the similarity of immune cell infiltration in the myocardium and skeletal muscle could also in turn suggest that immune system processes may play a role in the process of myocardial injury in dermatomyositis.

We finally identified 4 common hub-genes related to the immune process of myocarditis and DM: IFIT3, OAS3, ISG15, and RSAD2. Gene function enrichment analysis showed these genes were mainly enriched in type I interferon (IFN) signaling pathway, cellular response to type I interferon, and response to type I interferon. The maladaptive immune response created by type I IFN signaling is upregulated in many autoimmune diseases such as systemic lupus erythematosus (SLE), rheumatoid arthritis, Sjogren’s syndrome, and systemic sclerosis [37–39]. Several previous studies have confirmed that the type I IFN signaling pathway plays a prominent role in DM, and the expression levels of it is associated with DM activity [40–45]. Cassius et al. reported type I interferon signature was also to be highly expressed in the MDA5 + DM subtype [43]. In fact, a recent study suggests that inhibition of the type I IFN signaling pathway may reduce cardiovascular risk in SLE patients [46].

Given that immune cells play an essential role in the process of myocardial injury in DM, we sought to investigate the infiltration of immune cells in both DM and myocarditis patients. We found that T cells and macrophages comprise the majority of infiltrated immune cells in the skeletal muscle of DM, which is consistent with a recent study [47]. Furthermore, we observed an increase in both M1 and M2 macrophages in DM patients compared to healthy controls. Prior studies also revealed macrophages infiltration in the muscle is involved in the development and progression of DM [48, 49] and is associated with disease severity [50]. Increasing evidence showed that immune cell infiltration in the myocardium has adverse effect on heart function recently [51–53]. In the myocardium, macrophages are one of the most important cardiac immune cells and the central regulator of immune systems. In the past, macrophages were classified into M1 and M2 types by their surface molecules. Further, research indicated that M1 macrophages have a pro-inflammatory phenotype with anti-pathogen activity while M2 macrophages promote anti-inflammatory effects and tissue repair responses [54]. In animal models of experimental autoimmune myocarditis and viral myocarditis, the acute phase of myocarditis is dominated by pro-inflammatory macrophages, while the chronic phase is dominated by M2 macrophages [55–57]. The activation of the autoimmune system will eventually lead to excessive accumulation and transformation of macrophages, resulting in myocardial inflammation and fibrosis [58, 59]. In this study, the M2 macrophages of myocarditis patients increased while M1 macrophages were not statistically different from normal controls, which may be due to the fact that myocardium specimens in the selected dataset were in the chronic phase of myocarditis. Considering the similarities of immune cell infiltration in the myocardium and skeletal muscle, we speculated that the disorder in macrophages might play a potentially significant role in the process of myocardial injury in DM.

Previous studies have shown that miRNAs can be used as markers in a variety of cardiovascular diseases [60]. miR-146a-5p is an important regulator of the immune response and inflammation [61, 62] and is abundant in immune cells and the heart [63, 64]. It has been implicated in cardiac hypertrophy, ischemia/reperfusion injury, peripartum cardiomyopathy, doxorubicin toxicity, diabetic cardiomyopathy, and atherosclerosis [65–70]. The increased presence of circulating miR-146a-5p has been reported in patients with spontaneous coronary artery dissection, aortic dissection, and acute coronary syndromes [71–73]. Our study found that serum miR-146a-5p was significantly elevated in DM patients with myocardial injury than without myocardial injury, suggesting the potential of miR-146a-5p as a biomarker for assessing myocardial injury in DM.

There were certain limitations in our study. First, our study was based on bioinformatics analysis from public datasets, which may not fully reflect the actual situation. Secondly, due to the difficulty of obtaining cardiac samples from DM patients, we analyzed the gene sets of myocarditis and DM separately. Further in vitro and in vivo experiments are needed to confirm the role of common hub-genes in DM with myocardial injury. Thirdly, we searched only one dataset containing myocardial specimens in myocarditis patients, so we used murine myocardium for our subsequent validation. Finally, a larger sample size will be needed in future studies to verify the role of miR-146a-5p as a biomarker predicting myocardial injury in DM.

## Conclusion

Our study identified 4 common hub-genes related to the immune system process of myocarditis. We speculated that these genes may play a role in the process of myocardial injury in DM. Serum miR-146a-5p could be a potential biomarker to predict myocardial injury in DM.

## Data Availability

The data used and/or analyzed in the current study are available from the corresponding author on reasonable request.

## Abbreviations

DM: Dermatomyositis
IIM: Idiopathic inflammatory myopathy
NCBI-GEO: National Center Biotechnology Information Gene Expression Omnibus
PCA: Principal component analysis
WGCNA: Weighted gene co-expression network analysis
TOM: Topological overlap matrix
GS: Gene significance
MM: Module membership
STRING: Search Tool for the Retrieval of Interacting Genes
FC: Fold-change
LGE: Late gadolinium enhancement
DEGs: Differentially expressed genes
PPI: Protein-protein interaction
GO: Gene ontology
GSEA: Gene function enrichment analysis
IFN: Type I interferon
